# Poor Vitamin Status is Associated with Skeletal Muscle Loss and Mucositis in Head and Neck Cancer Patients

**DOI:** 10.3390/nu10091236

**Published:** 2018-09-05

**Authors:** Sara Nejatinamini, Brock J. Debenham, Robin D. Clugston, Asifa Mawani, Matthew Parliament, Wendy V. Wismer, Vera C. Mazurak

**Affiliations:** 1 Department of Agricultural, Food and Nutritional Science, University of Alberta, Edmonton, AB T6G 2E1, Canada; nejatina@ualberta.ca (S.N.); wwismer@ualberta.ca (W.V.W.); 2 Department of Oncology, University of Alberta, Edmonton, AB T6G 1Z2, Canada; Brock.Debenham@albertahealthservices.ca (B.J.D.); Asifa.Mawani@albertahealthservices.ca (A.M.); Matthew.Parliament@albertahealthservices.ca (M.P.); 3 Department of Physiology, University of Alberta, Edmonton, AB T6G 2H7, Canada; clugston@ualberta.ca

**Keywords:** head and neck cancer, cancer treatment, vitamin status, mucositis, skeletal muscle mass, body composition

## Abstract

Mucositis and muscle wasting are two common toxicity effects of cancer treatment in head and neck cancer (HNC). There is limited data evaluating cancer treatment toxicities in relation to vitamin status. This study aimed to assess changes in vitamin status during HNC treatment in relation to body composition, inflammation and mucositis. In this prospective cohort study, dietary intakes (3-day food record), plasma levels of vitamins and C-reactive protein (CRP) were assessed at baseline (at diagnosis) and post-treatment (after 6–8 weeks of radiation therapy with or without chemotherapy). Computed tomography images were used to quantify body composition. Mucositis information was collected from health records of patients. Twenty-eight HNC patients (age 60 ± 10 years) completed both study time points. Patients who developed mucositis had significantly lower dietary intake of vitamins and plasma 25-hydroxy vitamin D (25-OHD) and *all-trans* retinol levels (*p* < 0.02). Patients lost a considerable amount of muscle mass (3.4 kg) and fat mass (3.6 kg) over the course of treatment. There was a trend toward greater muscle loss in patients with 25-OHD < 50 nmol/L compared to patients with 25-OHD ≥ 50 nmol/L (*p* = 0.07). A significant negative correlation was found between plasma *all-trans* retinol and CRP level at the end of treatment (*p* = 0.03). Poor vitamin status could be a contributing factor in developing treatment-induced toxicities.

## 1. Introduction

The treatment of head and neck cancer (HNC) has evolved over the last several decades, with an increased emphasis placed on multimodality management. Despite advances in the management of HNC, treatment-induced toxicities that compromise dietary intake and nutritional status remain a common complication in HNC patients [1]. Previous studies have suggested that at diagnosis, 42–77% of HNC cancer patients experience malnutrition which is exacerbated over the course of cancer treatment [2]. This is clinically relevant, because poor nutritional status in cancer patients contributes to reduced immune function, increases treatment toxicities and diminishes the treatment response [3].

Weight loss during and after treatment is frequently noted among HNC patients. Traditionally, weight loss has been used to identify patients with cancer who are at risk for malnutrition [4,5]. However, muscle loss occurs in patients who may not be losing weight; therefore, measures of specific body components are required [6,7,8]. Assessment of cancer related malnutrition aims to identify and measure metabolic derangements and muscle depletion. Low muscle mass prior to treatment and muscle loss that occurred during treatment is associated with poor outcomes including poorer response to treatment and decreased survival [2,9]. Therefore, direct measures to quantify muscle loss and adipose tissue alterations are required; however only one study has precisely assessed body composition changes during treatment for head and neck cancer [10]. 

Oral mucositis in HNC patients undergoing radiotherapy with or without chemotherapy represents one of the most debilitating toxicities that affects quality of life and results in a high rate of hospitalization and treatment interruptions. There is limited data regarding an association between vitamin deficiencies and the development of mucositis [11]. Poor vitamin status in people with cancer has several possible causes, including unbalanced dietary intake, altered metabolism, adverse effects of treatment and inflammation. Deficiencies in certain vitamins correlate with systemic inflammation assessed by C-reactive protein (CRP) in cancer patients [12]. Understanding the role of vitamin status in mucositis development and correcting vitamin deficiencies before starting cancer treatment may prevent mucositis while enabling patients to receive the most appropriate treatment for their cancer. 

There is currently a low level of evidence regarding vitamin status in cancer patients and the European Society for Clinical Nutrition and Metabolism (ESPEN) recommends assessment of micronutrients in relation to oncological outcome as an understudied research area [13]. While the number of vitamin deficiencies in the HNC cancer population may be numerous, the focus of this research was vitamins A, D, E, folate and B_12_ because we have previously reported low dietary intake of these vitamins [14]. It was hypothesized that patients with low intakes and plasma levels of vitamins would experience greater loss of muscle and be more likely to develop mucositis as toxicity events during treatment for head and neck cancer. The objective of the present study was to investigate how vitamin status prior to and after cancer treatment in patients with HNC relates to body composition, mucositis and systemic inflammation. Further, we investigated whether baseline dietary intakes and plasma level of vitamins were related to the severity of mucositis and muscle loss.

## 2. Materials and Methods 

### 2.1. Study Population

This prospective cohort study was conducted at the Cross Cancer Institute, Edmonton, Canada. All subjects provided written informed consent prior to participation in the study. The study was conducted in accordance with the Declaration of Helsinki and the protocol was approved by the Health Research Ethics Board of Alberta-Cancer Committee (ethics number: 25852). Patients diagnosed with HNC that underwent radiation therapy, with or without chemotherapy, were invited to participate in the study. Eligibility criteria included pathologically confirmed squamous cell carcinoma of the oral cavity, pharynx, and larynx, with no history of a recurrent disease. Patients were excluded from the study if they were taking steroids or receiving palliative treatment. Patients who were unable to understand and speak English were excluded from the study. Thirty patients with HNC were enrolled in the study. Data were collected at diagnosis and prior to starting treatment (baseline) and at the completion of 4–6 weeks of radiotherapy with or without chemotherapy (post-treatment).

### 2.2. Dietary Intake

A trained researcher instructed patients on completion of the three-day dietary records collected at both baseline and post-treatment. The Canadian Nutrient File Database Analysis of the Food Processor II Nutrient Analysis ProgramTM (version 9: Esha Research, Salem, Oregon, OR, USA) was used to analyze dietary records and calculate the amount of calorie, protein and vitamin intake. Since vitamin intake at level of Canadian Recommended Dietary Allowance (RDA) is recommended by ESPEN for cancer patients [13], in this study vitamin intake was compared to the RDA for each vitamin to determine the degree to which the RDA that was met by patients on average. Participants were asked to complete a questionnaire about their micronutrient supplement intake.

### 2.3. Anthropometric and Body Composition Measurement

At both time points, body weight was measured without shoes and with light clothes by using a calibrated digital scale and recorded to the nearest 0.1 kg. Height was measured on a stadiometer and recorded to the nearest 0.1 cm at both time points. Body mass index (BMI) was calculated as weight in kilograms divided by height in meters squared at each time point (kg/m^2^). 

Body composition was assessed using computed tomography (CT) images taken for diagnostic purposes at baseline and after completion of the treatment (interval between 2 CT scans ~ 6 months). Seventeen patients had CT images at both time points of study. The 3rd lumbar vertebrae (L3) level was chosen as a landmark because of its high correlation to whole body muscle mass [15,16]. Images were analyzed using Slice-O-Matic software (Slice-O-Matic version 4.3, TomoVision, Magog, QC, Canada) to determine the adipose tissue and skeletal muscle cross-sectional areas (cm^2^) and muscle attenuation at L3 as previously described [17]. Muscle and adipose areas were normalized for height in meters squared (m^2^) and reported as the skeletal muscle index, visceral adipose index and subcutaneous adipose index; (cm^2^/m^2^). Whole body skeletal muscle and adipose tissue were calculated in conventional units using a regression formula: Whole body skeletal muscle mass = 0.166 × (skeletal muscle > 5 cm higher than L4 to L5 (cm^2^)) + 2.142; whole body adipose tissue mass = 0.068 × (adipose tissue > 5 cm higher than L4 to L5 (cm^2^)) + 4.142 [16].

### 2.4. Plasma Vitamins and CRP Measurement

Blood samples were taken at baseline and post-treatment, plasma was separated by centrifugation and aliquots were stored at −80 °C. Plasma folate and plasma holotranscobalamin (holoTC; the metabolically active portion of vitamin B_12_) levels were assessed using the AXSYM analyzer (Abbott Laboratories, Abbott Park, IL, USA) as per manufacturer’s instructions. For plasma folate level, we used a defined cutoff of <7 nmol/L for deficiency [18] and >46 nmol/L for above the normal range [19]. The reference value used for normal holoTC was 35 to 140 pmol/L [20]. Quantification of plasma 25-hydroxy vitamin D (25-OHD) level was determined by liquid chromatography-tandem mass spectrometry (LC-MS/MS) [21]. Mass detection was carried out with an API 5000 (AB SCIEX, Toronto, Canada). The reference range for sufficient vitamin D status was determined as >75 nmol/L [22]. *All-trans* retinol and α-tocopherol levels were analyzed by high-performance liquid chromatography (HPLC) Agilent 1200 HPLC system (Agilent Technologies, Santa Clara, CA) following hexane extraction from plasma using established protocols [23,24]. Plasma levels of *all-trans* retinol ≤0.70 μmol/L and α-tocopherol ≤12 μmol/L were used as deficiency cut off points [25,26]. 

CRP was determined using the CRPH enzyme-linked immunosorbent assay (Synchron LX system; Beckman Coulter, Inc., Fullerton, CA, USA). CRP assay functional sensitivity is estimated to be ≤0.18 mg/L which is defined by the lowest concentration that can be determined with *CV* = 20%.

### 2.5. Mucositis

Information regarding mucositis of the upper aerodigestive tract caused by cancer treatment was collected from health records of patients. Mucositis was graded by a nurse using the National Cancer Institute Common Terminology Criteria for Adverse Events (CTCAE v3.0). These scales range from 1–5, which are defined as follows: Grade 1 erythema of mucosa, grade 2 patchy ulcerations, grade 3 confluent ulcerations, grade 4 tissue necrosis, and grade 5 death. Patients with score of 1 or 0 were categorized as having no mucositis while patients with score 2 or higher were categorized as having mucositis. The most severe grade among several serial assessments was taken as final grade.

### 2.6. Statistical Analyses

Mean ± standard deviation was reported for continuous data; frequency and proportions were reported for categorical data. A paired sample *t*-test was used to compare the dietary intake, plasma vitamin levels and body composition between baseline and post-treatment. Pearson’s correlations were reported to assess the correlation of CRP level with skeletal muscle mass and plasma vitamin levels. Multiple linear regression analysis was performed to determine the correlation between skeletal muscle mass and possible independent variables. An independent sample *t*-test was used to compare differences in dietary intake and vitamin status between the mucositis and non-mucositis groups. Chi-square test used to compare mucositis prevalence in two cancer treatment arms (chemoradiotherapy vs radiotherapy alone or with surgery). All statistical analyses were performed using SPSS software (version 20 for Windows, IBM Corp., Armonk, NY, USA) and statistical significance was set at *p* < 0.05 in 2-tailed tests.

## 3. Results

### 3.1. Participant Characteristics

Baseline characteristics of the participants (*n* = 28) are shown in Table 1. The majority of subjects were male (82%). Mean age was 60.3 ± 10.8 years and mean BMI was 28.3 ± 5.6 kg/m^2^. The majority of patients had a locally advanced primary tumor in the pharynx (50%), and 82% had stage III or IV cancer.

### 3.2. Dietary Intake and Plasma Level of Vitamins

Analysis of dietary intake revealed no significant differences in energy and protein intakes from baseline to post-treatment (Table 2). However, both calorie and protein intakes were below the minimum range recommended by ESPEN at post-treatment. Patients failed to meet the RDAs for vitamins D, E, and folate at both time points of study. Dietary intakes of vitamin D increased from baseline to post-treatment (*p* = 0.04), although this had little effect on measured plasma level which remained stable during the study. The majority of patients were vitamin D deficient (<50 nmol/L) or insufficient (50–75 nmol/L). Only 2 patients had a circulating 25-OHD level that would be considered sufficient (>75 nmol/L) at both time points. Although vitamin A intake was higher than recommendations at both baseline and post-treatment, mean plasma *all-trans* retinol concentrations decreased significantly from baseline to post-treatment (*p* = 0.008) as the percent of patients with an insufficient level of retinol (<0.7 umol/L) increased from 4% at baseline to 46% at post-treatment. There were no significant changes in plasma concentrations of α-tocopherol nor folate during the cancer treatment. During the course of treatment, plasma level of active vitamin B_12_ increased significantly (*p* = 0.004).

### 3.3. Body Composition and Related Factors

Body weight declined considerably over the course of treatment with an average percent weight loss of −7.1 ± 3.9 (ranging from 1.7 to −14.2; Table 3). Approximately half of this loss was attributed specifically to muscle loss (3.4 kg) with an average loss of 12.6 ± 8.7% of their muscle volume and the other half could be explained by 3.6 kg fat loss. Patients experienced a significant decrease in both visceral and subcutaneous adipose tissue. Muscle attenuation decreased significantly over the course of treatment (*p* = 0.004). Patients with higher BMI at baseline lost more weight (*r* = −0.43, *p* = 0.02) and skeletal muscle mass (*r* = −0.53, *p* = 0.02) during treatment (Table 3).

There was a trend toward greater muscle loss in patients with 25-OHD < 50 nmol/L compared to patients with 25-OHD ≥ 50 nmol/L (−15.4% vs. −7.6%; *p* = 0.07). After controlling for age and sex, higher plasma 25-OHD was associated with greater muscle cross-sectional area at baseline and post-treatment (Table 4). This correlation remained significant after considering CRP, stage of disease and type of treatment in the regression model.

### 3.4. Plasma CRP Levels

There was a significant increase in CRP over the course of treatment for all patients (6.7 ± 9.9 and 15.3 ± 16.7 mg/L; *p* = 0.01). Plasma *all-trans* retinol level was negatively correlated with CRP level (*r* = −0.57, *p*-value = 0.03) at the post-treatment time point. Patients with higher level of CRP had lower skeletal muscle mass at baseline and post-treatment (*r* = −0.5, *p* = 0.01; *r* = −0.51, *p* = 0.04, respectively).

### 3.5. Mucositis

The occurrence of moderate to severe mucositis (score 2 or higher) was observed in 52% patients at some point during the treatment. Patients with mucositis compared to those without mucositis had lower dietary intakes of vitamins D, E, folate, and B_12_ at baseline (Table 5). Patients with mucositis had significantly lower plasma *all-trans* retinol and 25-OHD at baseline compared to patients without mucositis (Table 5).

Patients with mucositis compared to those without mucositis had higher BMI (30.3 ± 6.6 vs. 26.2 ± 3.6 kg/m^2^; *p* = 0.04) at baseline. Patients who received chemoradiotherapy had significantly higher prevalence of mucositis compared to patients who underwent radiotherapy alone or with surgery (*p* = 0.001). Weight loss during the course of treatment was higher in those who developed mucositis compared to those without mucositis (9.0 ± 3.2 and 5.1 ± 3.7%, *p* = 0.009, respectively) even though there were no significant differences in energy and protein intake between groups. Moreover, there was a trend toward higher skeletal muscle loss in patients with mucositis (−14.7 ± 8.2 vs. −7.5 ± 8.6%, *p* = 0.07).

## 4. Discussion

This study reveals an association between vitamin status, muscle mass and mucositis in HNC patients undergoing treatment. Over the course of treatment, HNC patients lose a considerable amount of weight which is explained by equal losses of muscle and fat. We also report a high prevalence of Vitamin D deficiency and insufficiency among HNC cancer patients. Patients who developed mucositis had poor micronutrient intake at baseline and lower plasma vitamin D and all-trans retinol level compared to patients without mucositis over the study time points. The decline in plasma concentration of all-trans retinol over the course of treatment related to elevated plasma CRP level.

Negative energy and protein balance are important factors that contribute to loss of body weight and lean mass in patients with cancer. The patients in this study lost 7.1 kg of weight and 3.4 kg skeletal muscle during cancer treatment. Low dietary intake due to treatment-related nutrition impact symptoms may have been one of the main contributing factors for muscle loss in HNC patients. Dietary intake of our patients was markedly lower than the ESPEN recommendations of 25 to 30 kcal/kg and 1 to 1.5 g protein/kg body weight by the end of treatment. However, the actual intake of calorie and protein required to prevent muscle loss in HNC patients has not yet been determined. In a study by Jager-Wittenaar et al., 2011, protein intake >1.7 g protein/kg body weight was suggested as the optimal protein intake to reduce weight and muscle loss in HNC patients during cancer treatment [27]. In addition to low dietary intake, inflammation could exacerbate muscle loss during cancer treatment. The negative correlation between CRP and muscle mass was observed in the current study at both baseline and post-treatment. Similarly, in a study of 471 cancer patients with solid tumors, those with CRP >10 mg/L had less muscle and lost more muscle during the disease trajectory [28]. Understanding this association might provide a better illustration of the mechanism of muscle loss in HNC patients, therefore interventions that target inflammation may provide a benefit to attenuate muscle loss during treatment for HNC.

Our results suggest that HNC patients are not meeting recommended intakes for vitamin D, E, and folate at diagnosis, nor after completion of cancer treatment. Furthermore, we observed that habitual diets of poor vitamin intake may increase risk for toxicities during cancer treatment. There are few studies which have evaluated vitamin intakes in relation to the development of chemotherapy toxicities in cancer patients [29,30]. Our study suggests development of mucositis may relate to plasma levels of 25-OHD and all-trans retinol in patients with HNC. A prospective observational study in cancer patients reported that vitamin D deficiency was associated with a higher severity of treatment-related toxicities in patients receiving pelvic radiotherapy [31]. However, in another study by Kitchen et al., 2015 in a mixed group of cancer patients, no relationship was observed between 25-OHD level and toxicities [32]. This discrepancy may be the result of including a heterogeneous group of cancer patients undergoing different types of treatment and experiencing a variety of symptoms in the latter study. The involvement of vitamin D in development of mucositis could be explained by its crucial role in maintaining mucosal barrier homeostasis and modulation of immune responses.

This study is the first that we know of to prospectively evaluate the relationship between circulating 25-OHD concentrations and longitudinal changes in muscle mass in HNC cancer patients. The current study revealed that plasma 25-OHD level was related to skeletal muscle mass even after considering confounding factors in the multiple regression model. Patients with 25-OHD levels < 50 nmol/L lost twice the amount of skeletal muscle during cancer treatment, but this trend did not reach statistical significance. A larger sample size is required to determine this in future studies. Our results are in line with the findings from other studies in which poor vitamin D status was prospectively associated with greater appendicular skeletal muscle mass loss in older adults [33,34]. The underlying mechanisms include both an indirect role of vitamin D through calcium and phosphate and a direct role via vitamin D receptor activation and regulation of the transcription of several genes involved in protein synthesis, differentiation and proliferation of muscle cells [35].

Plasma levels of *all-trans* retinol decreased significantly over the course of treatment, which is in agreement with other reports reporting low circulating concentrations of retinol in patients with cancer [12]. Retinyl esters serve as a hepatic storage form of retinol to provide requirements over a long period of time. Intake of retinol by patients was higher than the recommended levels at baseline and post-treatment so the acute reduction in circulating retinol during cancer treatment is not likely due to low retinol intake in the diet. Decreased synthesis of proteins involved in retinol transport, retinol binding protein, and transthyretin, in response to the acute-phase reaction to inflammation could collectively contribute to this [36]. The results of our study are in accordance with other studies, where lower serum retinol was associated with plasma level of CRP in HNC patients [12]. The clinical relevance of this finding may reflect severity of acute phase reaction in HNC patients.

Our study has a number of strengths including a prospective study design, assessing vitamin status through both dietary intake and plasma level, using CT scan images to assess body composition as a gold standard method in homogenous group of patients. Limitations of this study included the small sample size and also the lack of data regarding smoking and alcohol intake of patients which may affect the circulating level of vitamins. Moreover, plasma levels of vitamins may not be the most reliable measure of certain vitamins status such as vitamin B_12_.

## 5. Conclusions

In conclusion, patients who have diets containing low vitamin content, low plasma levels of 25-OHD and/or all-trans retinol are more likely to experience mucositis during cancer treatment. Therefore, measurement of plasma levels of *all-trans* retinol and 25-OHD in HNC at baseline could identify those patients at risk for mucositis development, which needs further investigation. Our study confirms a correlation between plasma 25-OHD level with skeletal muscle mass as well as a tendency to lose more muscle in patients with lower 25-OHD concentration. Plasma *all-trans* retinol decreased significantly during cancer treatment which could be an indicator of the severity of inflammation during cancer treatment. Further research is needed to fully characterize the effects of vitamin status on treatment-induced toxicities in homogenous groups of patients with cancer.

## Figures and Tables

**Table 1:** Baseline characteristics of patients (*n* = 28).

Characteristics	Value
Sex, male, *n* (%)	23 (82)
Age (years), mean (SD)	60.3 (10.8)
BMI * (kg/m^2^), mean (SD)	28.3 (5.6)
Tumor classification **, *n* (%)	
I	1 (4)
II	4 (14)
III	19 (68)
IV	4 (14)
Mode of treatment, *n* (%) Radiotherapy	6 (22)
Chemo-radiotherapy	20 (71)
Radiotherapy + Surgery	2(7)
Tumor site, *n* (%)	
Lip/oral cavity	11 (39)
Pharynx	14 (50)
Larynx	3 (11)

* BMI: Body mass index; ** American Joint Committee on Cancer (AJCC) Staging 7th Edition 2010 (version 01.04.00).

**Table 2:** Dietary intake and plasma level of vitamins at baseline and post-treatment.

	Baseline	Post-Treatment	*p*-Value
Calories, kcal/kg BW */day	23.1 ± 8.3	19.7 ± 9.8	0.17
Protein, g/kg BW/day	1.0 ± 0.4	0.8 ± 0.4	0.10
**Dietary intake of vitamins**			
Vitamin A, (%RDA) **	158 ± 32	124 ± 14	0.32
Vitamin D, (%RDA)	36 ± 5.6	53 ± 6.1	0.04
Vitamin E, (%RDA)	41 ± 7.0	74 ± 19.0	0.11
Folate, (%RDA)	72 ± 9.4	75 ± 10.0	0.80
Vitamin B_12_, (%RDA)	255 ± 50	234 ± 25	0.72
**Plasma level of vitamins**			
*All-trans* retinol, µmol/l	0.86 ± 0.2	0.69 ± 0.2	0.008
25-OHD, nmol/l	55.1 ± 17.7	54.5 ± 18.9	0.78
α-tocopherol, µmol/l	9.5 ± 2.8	9.9 ± 4.0	0.78
Folate, nmol/l	31.2 ± 14.0	27.8 ± 8.3	0.19
HoloTC ***, pmol/l	53.9 ± 14.0	74.7 ± 8.3	0.004

Data presented as mean ± SD. RDA: Recommended Dietary Allowance; * BW: body weight; ** %RDA: the proportion of the Canadian RDA (Recommended Dietary Allowance) that was met by patients on average; *** HoloTC: Holotranscobalamin.

**Table 3:** Anthropometric variables of patients at baseline and post-treatment.

Variables	Baseline	Post-Treatment	*p*-Value
Body weight, kg	87.2 ± 3.3	80.7 ± 2.8	<0.001
Muscle area (cm^2^)	159.2 ± 41.7	137.8 ± 34.7	<0.001
Skeletal Muscle index (cm^2^/m^2^)	52.6 ± 11.1	45.5 ± 9.1	<0.001
Estimated whole body muscle (kg)	28.5 ± 5.4	25 ± 6.3	0.002
Muscle attenuation (HU) *	31.6 ± 9.0	26.1 ± 6.9	0.004
Visceral adipose tissue (cm^2^)	169.6 ± 74.9	131.9 ± 77.7	0.001
Visceral adipose index (cm^2^/m^2^)	57.3 ± 25.9	44.8 ± 27.5	0.001
Subcutaneous adipose tissue (cm^2^)	226.8 ± 153	170.1 ± 112.1	0.01
Subcutaneous adipose index(cm^2^/m^2^)	77.7 ± 54.8	58.6 ± 40.9	0.01
Total adipose tissue (cm^2^)	408 ± 194.4	313.3 ± 165.9	0.002
Estimated whole body fat mass (kg)	28.3 ± 8.1	24.7 ± 6.9	0.002

Data presented as mean ± SD. * HU: Hounsfield unit.

**Table 4:** Multiple regression analysis with skeletal muscle mass as the dependent variable and sex, age, 25-OHD as independent variable.

Time Point	Variable	β	Standard Error	*p*-Value
Baseline *				
	Sex	64.1	0.70	<0.0001
	Age	−1.4	−0.39	0.01
	25-OHD (nmol/L)	0.74	0.36	0.01
Post-treatment **				
	Sex	57.8	0.78	<0.0001
	Age	−0.84	−0.28	0.07
	25-OHD (nmol/L)	0.63	0.37	0.02

25-OHD: 25-hydroxy vitamin D; * Baseline regression equation: *F*(3,18) = 14.76, *p* < 0.0001, with an *R*^2^ of 0.711; ** Post-treatment regression equation: *F*(3,12) = 14.28, *p* < 0.0001, with an *R*^2^ of 0.781.

**Table 5:** Baseline dietary intake and plasma level of vitamins in patients based on mucositis status.

	No Mucositis	Mucositis	*p*-Value
Calorie, kcal/kg BW */day	24.2 ± 5.6	21.7 ± 9.7	0.43
Protein, g/kg BW/day	1.05 ± 0.28	0.98 ± 0.46	0.63
**Dietary intake of vitamins**			
Vitamin A, IU **/day	5403 ± 672	3635 ± 1056	0.16
Vitamin D, IU/day	339 ± 184	140 ± 89	0.002
Vitamin E, mg/day	10.7 ± 7.9	4.7 ± 2.8	0.013
Folate, mcg/day	368 ± 190	231 ± 147	0.04
Vitamin B_12_, mcg/day	6.3 ± 2.5	3.5 ± 2.2	0.01
**Plasma level of vitamins**			
*All-trans* retinol, umol/l	0.95 ± 0.15	0.77 ± 0.19	0.023
25-OHD, nmol/l	62.3 ± 14.0	47.2 ± 17.9	0.025
α-tocopherol, umol/l	9.5 ± 2.6	9.2 ± 2.9	0.78
Folate, nmol/l	34.5 ± 16.9	26.8 ± 8.3	0.16
HoloTC *** pmol/l	52.5 ± 19.7	54 ± 28.9	0.87

Data presented as mean ± SD; * BW: body weight; ** IU: international unit; *** HoloTC: Holotranscobalamin.
